# Application of a Patient Derived Xenograft Model for Predicative Study of Uterine Fibroid Disease

**DOI:** 10.1371/journal.pone.0142429

**Published:** 2015-11-20

**Authors:** Martin Fritsch, Nicole Schmidt, Ina Gröticke, Anna-Lena Frisk, Christopher S. Keator, Markus Koch, Ov D. Slayden

**Affiliations:** 1 Bayer Pharma AG, Global Drug Discovery, Berlin, Germany; 2 Oregon National Primate Research Center, Oregon Health & Science University, Beaverton, Oregon, United States of America; Queen's University, CANADA

## Abstract

Human uterine fibroids, benign tumors derived from the smooth muscle layers of the uterus, impose a major health burden to up to 50% of premenopausal women in their daily life. To improve our understanding of this disease, we developed and characterized a patient-derived xenograft model by subcutaneous transplantation of pieces of human uterine fibroid tissue into three different strains of severe combined immunodeficient mice. Engrafted uterine fibroid tissue preserved the classical morphology with interwoven bundles of smooth muscle cells and an abundant deposition of collagenous matrix, similar to uterine fibroids in situ. The grafts expressed both estrogen receptor 1 and progesterone receptor. Additionally, both receptors were up-regulated by estrogen treatment. Growth of the fibroid grafts was dependent on 17β-estradiol and progesterone supplementation at levels similar to women with the disease and was studied for up to 60 days at maximum. Co-treatment with the antiprogestin mifepristone reduced graft growth (four independent donors, p<0.0001 two-sided t-test), as did treatment with the mTOR inhibitor rapamycin (three independent donors, p<0.0001 two-sided t-test). This in vivo animal model preserves the main histological and functional characteristics of human uterine fibroids, is amenable to intervention by pharmacological treatment, and can thus serve as an adequate model for the development of novel therapies.

## Introduction

Human uterine fibroids (UFs; uterine leiomyoma) are benign tumors of the myometrial layers of the uterus. They represent a major and underestimated health burden for women with an incidence in black and white women age 35–49 at approximately 80% and 70%, respectively, in the United States [1]. In more than half of those patients the uterine fibroids are considered to be clinically relevant. UFs are mainly diagnosed when women present to their physician with irregular or excessive uterine bleeding, pelvic pain or pressure, or infertility [2]. In a European study, between 12% and 24% of all women were diagnosed for UFs when presenting with uterine bleeding symptoms [3]. In the United States, fibroids are cited to be the cause for over 50% of hysterectomies [4], and direct costs for their treatment has been estimated between 4 and 9 billion US $ [5].

Histologically, UFs consist of interwoven bundles of smooth muscle cells and areas of hyalinized stroma, which in electron microscopy appears as massive deposition of disordered extracellular matrix (ECM) [6]. Fibroid smooth muscle cells appear spindle-shaped, are arranged in whirl-like structures, and express markers of smooth muscle cells, including desmin and alpha-smooth muscle actin, as well as estrogen receptor alpha and progesterone receptor [7]. Uterine fibroids show low mitotic rates comparable to or intermediate between normal myometrial tissue and leiomyosarcoma [8]. In contrast to the surrounding myometrial tissue, UFs are hypoxic, but fail to show typical responses to hypoxia such as upregulation of HIF1alpha [9]. In patients with multiple UFs, both unicentric and multicentric development of the tumors has been demonstrated [10].

Approximately 30% of UFs, display karyotypic aberrations [11,12], but it remains unclear whether these genetic alterations cause fibroid formation, or are secondary events associated with the development of fibroid tumors. In general, growth of UFs requires endocrine support from the ovarian steroid hormones, 17β estradiol (E2) and progesterone (P4). Currently, there are few medical treatment options which in general target the normalization or reduction of female sex hormones E2 and P4. Symptomatic UFs are most often treated surgically (hysterectomy or myomectomy) or by uterine artery embolization [13].

Despite the fact that UFs represent more than 99% of all uterine smooth muscle cell tumors [14], the etiology of UFs is barely understood. Our understanding of UF disease and the development of medical therapies for UFs is severely hampered by the lack of in vivo models for this disease. One model, the EKER rat, is the only laboratory animal developing spontaneous uterine leiomyoma. The EKER rat is haploid deficient for the Tsc2 gene locuswhich, together with Tsc1, shows GTPase and tumor suppressor activity inactivating the mTOR pathway [15,16]. Mutations in the Tsc gene(s) results in uterine leiomyomata and renal cancer in murines [17]. In humans, mutations in the *TSC* gene(s) have been described to cause cysts and angiomyolipoma in the kidney, but not UFs in women [18]. The EKER rat model is limited by the very low incidence of the disease, which is only 36% for macroscopic lesions at 12 month of age. In addition, the developing uterine leiomyomas show relatively small amounts of collagenous connective tissue stroma [16]. Nevertheless, the EKER rat model was instrumental in demonstrating the effectiveness of rapamycin analogues in treating UFs [19].

A recent publication suggested that mutations in exon 2 of the *MED12 gene being* a subunit of the larger mediator complex integrating transcription factor activity controlling gene expression cause up to 70% of all UFs in women [20]. We therefore propose that the only suitable model system would display all of the potential genetic variability of human fibroids in women. By transplanting human fibroid tissues or cells into an immunodeficient mouse strain, patient-derived UF in vivo models can be generated, preserving the characteristics of the tumors. Some efforts had been taken in this direction; Hassan et al. transplanted human uterine tissue pieces s.c. into high-dose E2 supplemented severe combined immunodeficient (SCID) mice. However, the resulting xenografts showed only minimal growth even with additional adenoviral co-transfection of *VEGF*A and *COX2* [21]. A similar experiment was conducted by Tsuji et al. where the xenografts essentially showed no growth under E2 supplementation [22]. Ishikawa et al. transplanted pieces of UF tissue under the renal capsule of E2 and P4 supplemented mice. Under these conditions, tumors showed significant growth, but the number of grafts and their size was very restricted, and the surgery was rather demanding; making the method not suitable for large scale analysis [23].

In this study we demonstrate that UF and myometrium can be transplanted into immunodeficient SCID mice. Supplementing the mice with suitable doses of E2 and P4 supports the growth of subcutaneously transplanted human fibroids xenografts. The grafted tissue maintains characteristics typical of human fibroids in women. This model is very well suited for the evaluation of new treatment modalities and we show that otherwise established inhibitors of UF growth such as rapamycin and mifepristone inhibit growth of patient-derived xenografts.

## Materials and Methods

The studies reported herein were conducted independently at two sites, Bayer Pharma AG Berlin, and at the Oregon National Primate Research Center (ONPRC) in Portland Oregon.

### Human fibroid tissue xenograft

Uterine fibroid and myometrial tissue were obtained after prior review and approval of the studies by the respective institutional review board from the Charité University Hospital (ethics committee approval number EA4/023/05), the Auguste-Viktoria Hospital (ethics committee approval number Eth-0007/10) and the Hospital for Minimal Invasive Surgery (ethics committee approval number Eth-08/10) in Berlin and the Research Hospital at Oregon Health & Science University (OSHU) in Portland, Oregon. All samples were anonymized and written consent of the donors in accordance with the ethics committee guidelines of the respective hospitals was obtained. The tissue was transported within 24 hours to the laboratory in a suitable buffer (Viaspan®; Bristol-Myers Squibb) on ice. Tissue samples were cut aseptically into either small (2x2x2mm) or large (2x4x4mm) grafts using a razor blade, while keeping the tissue moist at all times. Mean weights of surplus tissue pieces not used for transplantation were taken as day 0 controls.

Immunodeficient mice (either CB17-SCID (inbred), ICR-SCID (outbred), SCID-beige (inbred), NOG SCID (inbred), NOD SCID (inbred) or genetically modified Rag2γC null mice; 4–6 month old, purchased from Taconic or Charles River Laboratories) were maintained under barrier conditions and used as recipients for fibroid transplantation. Studies carried out at the Oregon National Primate Center were done in accordance with the recommendation in the Guide for the Care and Use of Laboratory animals of the National Institutes of Health. The protocol was reviewed and approved by the ONPRC/OHSU Animal Care and Use Committee (IACUC approval # IS00000539). Animal experiments at Bayer AG were performed after review and approval by the LaGeSo (Berlin, for Bayer AG) under the permit number A 0333/11. The respective protocols include appropriate anesthesia and subsequent analgesia for surgical interventions and pain relief, respectively. Mice used in our experiments were either bought ovariectomized by the indicated breeders, or intact SCID mice were ovariectomized via a single dorsal incision in our animal facitlities. Ovariectomy took place 1 to 4 weeks before the animals were used in the respective experiment. Fibroid or myometrial tissue pieces, either eight small size or four large size tissue pieces, were transplanted s.c. ventrally into ovariectomized mice. During the same surgery, mice were supplemented s.c. in the neck with E2 and P4 pellets (Innovative Research of America, Sarasota, FL, USA). Unless otherwise indicated, the standard dose of E2 and P4 was 0.05mg/90d release and 25mg/60d release, respectively. During the experiment, the mice received standard chow and water ad libitum. BrdU (0.8mg/ml, SigmaAldrich) in drinking water was given for the last seven days of the experiment.

Xenograft growth was studied between 15 days and 60 days after graft inoculation, dependent on the individual experimental design (details are given in the text and figure legends). At the end of each experiment the mice were killed by terminal blood collection from the vena cava under general isoflurane anesthesia. Grafts were carefully prepared, freed from surrounding mouse tissue and weighted. Subsequently, grafts were either fixed in 4% (v/v) buffered paraformaldehyde, snap frozen in O.C.T. cryoembedding media (Sakura Finetek, Torrance CA) or frozen in liquid nitrogen. Unless otherwise indicated, the numbers of the tissue donors in the figures is only to discriminate between different donors; tissue donors in different figures are not identical.

### Histology

After fixation and routine dehydration, all tissue samples were embedded in paraffin, 4–6 μm thick sections were stained with hematoxylin-eosin for microscopical examination.

### Immunohistochemistry

Immunohistochemistry for *ESR1* gene product ERα, *PGR* gene product progesterone receptor (PR), and Ki-67 was conducted on OCT- embedded cryosections as previously described [24]. Briefly, cryosections (7 um) were mounted on SuperFrost Plus slides, fixed in 2% paraformaldehyde in PBS (pH 7.3; Sigma) for 10 min at room temperature (RT; ~ 23°C). Slides were rinsed and then incubated in a buffered solution containing glucose oxidase (1 U/mL), sodium azide (1 mM/L), and glucose (10 mM/L) for 45 min to inhibit endogenous peroxidase activity. The sections were treated with normal horse serum (20 min) and then with an endogenous biotin blocking solution (Vector Labs, Burlingame, CA, USA) for 30 min. The sections were incubated overnight at 4°C with mouse monoclonal antibodies directed against ERα (4 ug/mL; Clone 1D5; Thermo Fisher Scientific, Rockford, Ill, USA), PR (1 ug/mL; Ab-8; Thermo), or anti-Ki-67 antigen (Dako Corp., Carpinteria, CA, USA). Following incubation with primary antibody, the slides were rinsed in 0.075% BRIJ 35 (30% Stock; Sigma Chemicals, St. Louis, MO, USA) in PBS. The sections were incubated with blocking serum for 20 min and then with biotinylated anti-mouse IgG for 45 min at RT. The slides were rinsed again with PBS and reacted with an avidin-biotin peroxidase kit (Vector Labs) for 60 min (at RT) then rinsed in Tris buffer (pH 7.6). The slides were incubated for 15 min in 0.025% diaminobenzidine to visualize antibody-antigen complexes, which were further stabilized by incubating the slides in 0.026% osmium tetraoxide for 1 min (RT). Slides were lightly counterstained with hematoxylin, dehydrated in ethanol, cleared with xylene and mounted with Permount.

Immunohistochemistry for ERα and PR was viewed on a Zeiss AxioImager A.1 microscope (Carl Zeiss, Inc., Oberkochen, Germany) with planapochromatic lenses. Digital photomicrographs were captured with a Leica DFC 480 camera (Leica, Wetzlar, Germany). No post capture adjustments were made to the images; photomicrographic plates were cropped and annotated in Photoshop Creative Suite 4 (Adobe Systems, Seattle, WA, USA).

Immunohistochemistry for BrdU incorporation into DNA, Desmin for intermediate filament in smooth muscle, and Sirius Red stain for collagen (extracellular matrix) in the grafts was done on 4% formaldehyde-fixed and paraffin-embedded tissue. 4μm sections were mounted on Superfrost Plus slides and deparaffinized with xylol and descending concentrations of ethanol. For BrdU stain, the antigen was demasked by incubating the slides for 10min in 1N HCl on ice, followed by 30min in 2N HCl at 37°C and subsequently neutralized in 0.1M borate buffer and rinsed with distilled water. Endogeneous perodixidase and unspecific protein binding were blocked by commercially available kits (DAKO S2001 and X0909). Slides were incubated with anti-BrdU antibody (1:200; clone Bu20a, DAKO, Germany) for 30min and developed with a secondary HRP antibody and diaminobenzidine (EnVision, DAKO, Germany). For Desmin stain, antigen demasking was done in a microwave oven in antigen retrieval buffer (DAKO S1699). Slides in buffer were heated until cooking, and then incubated at 90W for 2-times seven minutes. After peroxidase and unspecific binding block as described above, slides were incubated with anti-Desmin antibody (1:100, clone D33, DAKO, Germany) and visualized as described above. Collagen deposition was stained with 0.1% Sirius red in a saturated aqueous solution of picric acid for 1h. Counterstain of cellular cytosol was done by 15 second incubation of the slides in 0.1% Fast Green FCF in 92% ethanol. Slides were rinsed with isopropanol. For all stains, slides were dehydrated in ethanol, cleared with xylene and mounted with Histomount. Slides were viewed with an Olympus BX61 microscope (Olympus, Hamburg, Germany) with planapochromatic lenses. Digital photomicrographs were taken with the Ariol^®^ system (Applied Imaging–Leica microsystems, San Jose, CA) attached to the microscope. No postcapture adjustments were made to the images.

### 17β Estradiol and Progesterone Steroid Assays in mouse plasma

E2 in Li-Heparin plasma from mice was determined using a LIA assay (IBL international, Hamburg, Germany). For determination of the unknown plasma concentrations, we used a self-prepared standard curve ranging from 0.05 to 9pg E2 on charcoal-stripped mouse plasma. E2 determinations of this assay were validated and shown to be essentially the same as for a LC/MS/MS method with deuterated E2 as a reference. P4 in mouse Li-Heparin plasma was quantified with a RIA assay (Coat-a-Count, Siemens, Germany) as described by the manufacturer using a self-prepared standard curve as a reference for quantification.

### Statistics

To analyze differences in graft growth between the different treatment groups, all graft weight data were log transformed. For further testing, we assumed identical variances for groups in each comparison. Significances are reported in the graphs as **** for p<0.0001, *** for p<0.001, ** for p<0.005, * for p<0.05 and ns for not significant. Details on the statistical tests performed are outlined in the respective figure legend.

## Results

### 17β Estradiol and Progesterone are indispensable to support uterine fibroid xenograft growth in SCID mice

**Fig 1:**
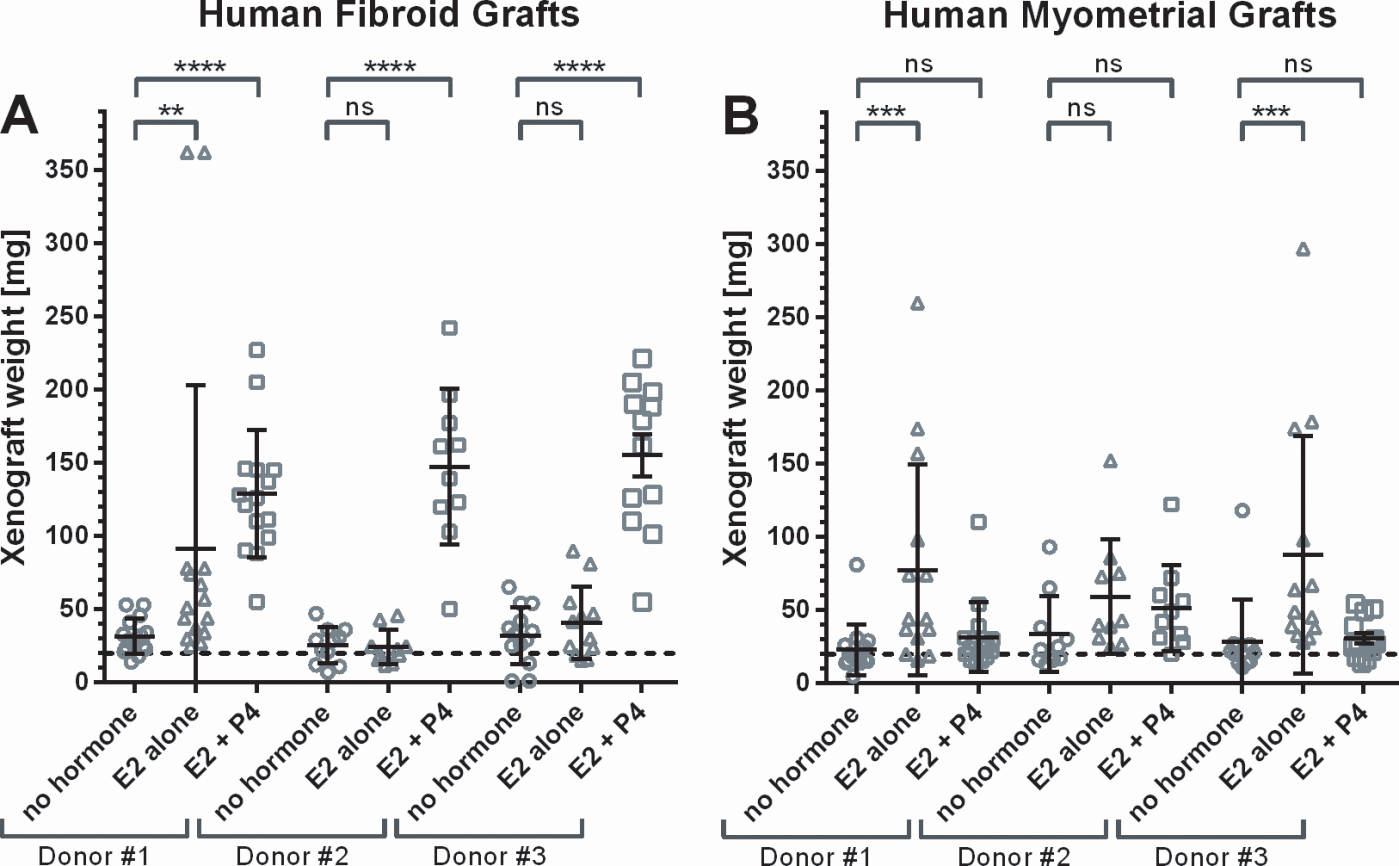
17β Estradiol and Progesterone support growth of fibroid xenografts. CB17-SCID mice were ovariectomized, and either not treated or supplemented with either 17β Estradiol (0.1mg/60d release pellets) alone, or 17β Estradiol in combination with progesterone (25mg/60d release pellets). Per mouse, four small grafts of each fibroid and myometrial tissue from one donor was transplanted. Between four to six mice were transplanted per donor and treatment group in this experiment (for details see S1 Table)The weight of the grafted fibroid tissue pieces at grafting is indicated with the dotted line. Mice were sacrificed after 60d, and grafts were weighted. Symbols indicate individual graft weights, together with group means ± s.e.m.

**Fig 2:**
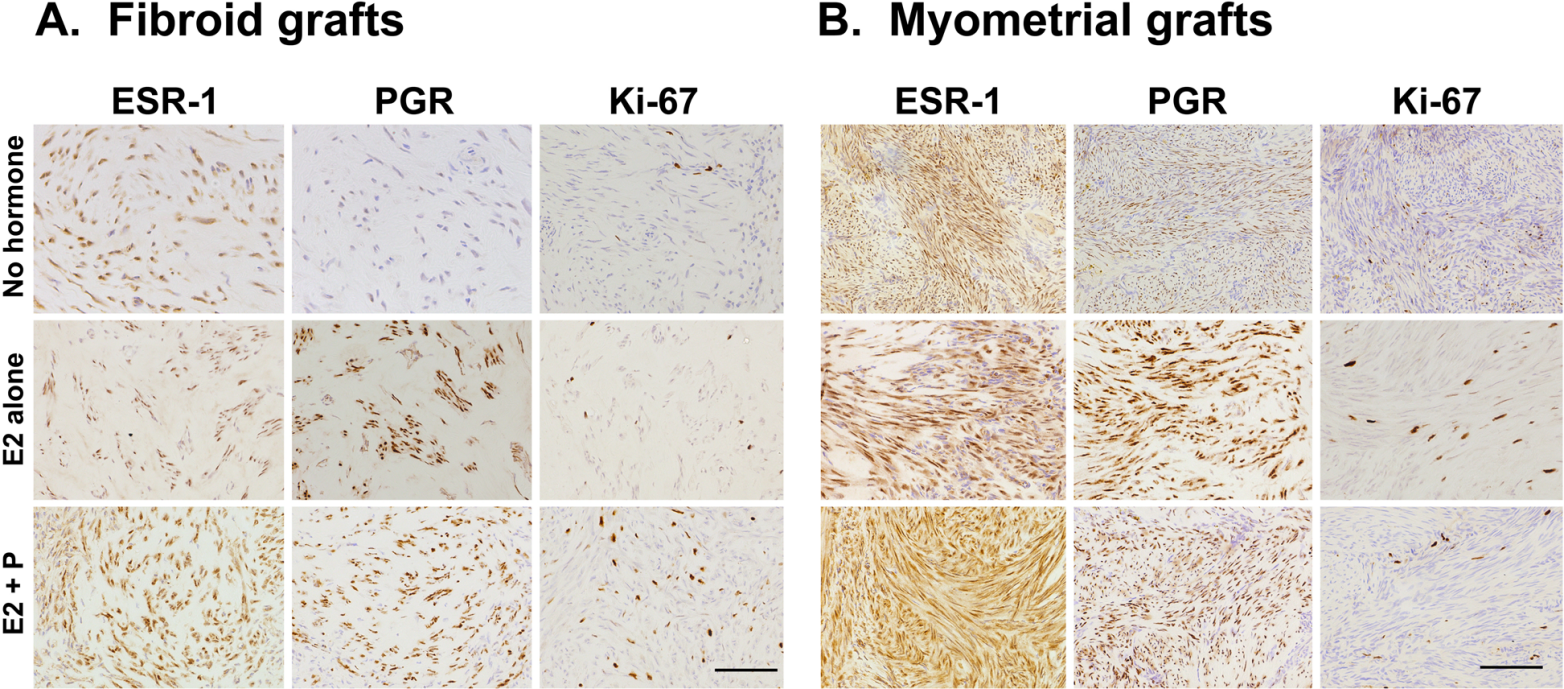
Immunohistochemistry of Estradiol receptor alpha, Progesterone receptor, and Ki67 in human fibroid grafts. Uterine fibroid and myometrial grafts from the mouse xenograft experiment shown in Fig 1 were stained for ERα, PR and Ki67.

Cell proliferation in fibroids in women is dependent on female sex steroids, and highest proliferation rates in fibroids are seen in the presence of 17β Estradiol (E2) and Progesterone (P4) in the secretory phase of the menstrual cycle [25,26,27]. We therefore transplanted primary human fibroid tissue and myometrial grafts in immunodeficient mice in the presence of E2 and P4. Fig 1 shows that reliable weight gain over 60 days in fibroid xenografts, but not myometrial grafts, is supported by E2 (0.1mg/60d release pellets) in combination with P4 (25mg/60d release pellets) as determined by final xenograft weight. E2 alone showed only weak stimulation of growth in some fibroid and myometrial grafts. In myometrial grafts, less growth is seen with E2 plus P4 supplementation than with E2 alone. Cell proliferation in grafts at the end of the experiment was visualized by Ki67 stain and showed strongest staining in fibroid grafts from mice supplemented with E2 plus P4, which was in accordance with the highest weight gains in these tissues (Fig 2). Since hormones exert their action via the respective hormone receptors, we analyzed the expression of the estrogen receptor alpha and progesterone receptor in the fibroid and myometrial grafts at the end of the experiment (Fig 2). The ERα and the PR remain expressed in fibroid as well as myometrial grafts under E2 alone as well as under E2 plus P4 treatment.

### E2 and P4 concentrations for optimal xenograft growth are in the same range as in the secretory phase of the female menstrual cycle

**Fig 3:**
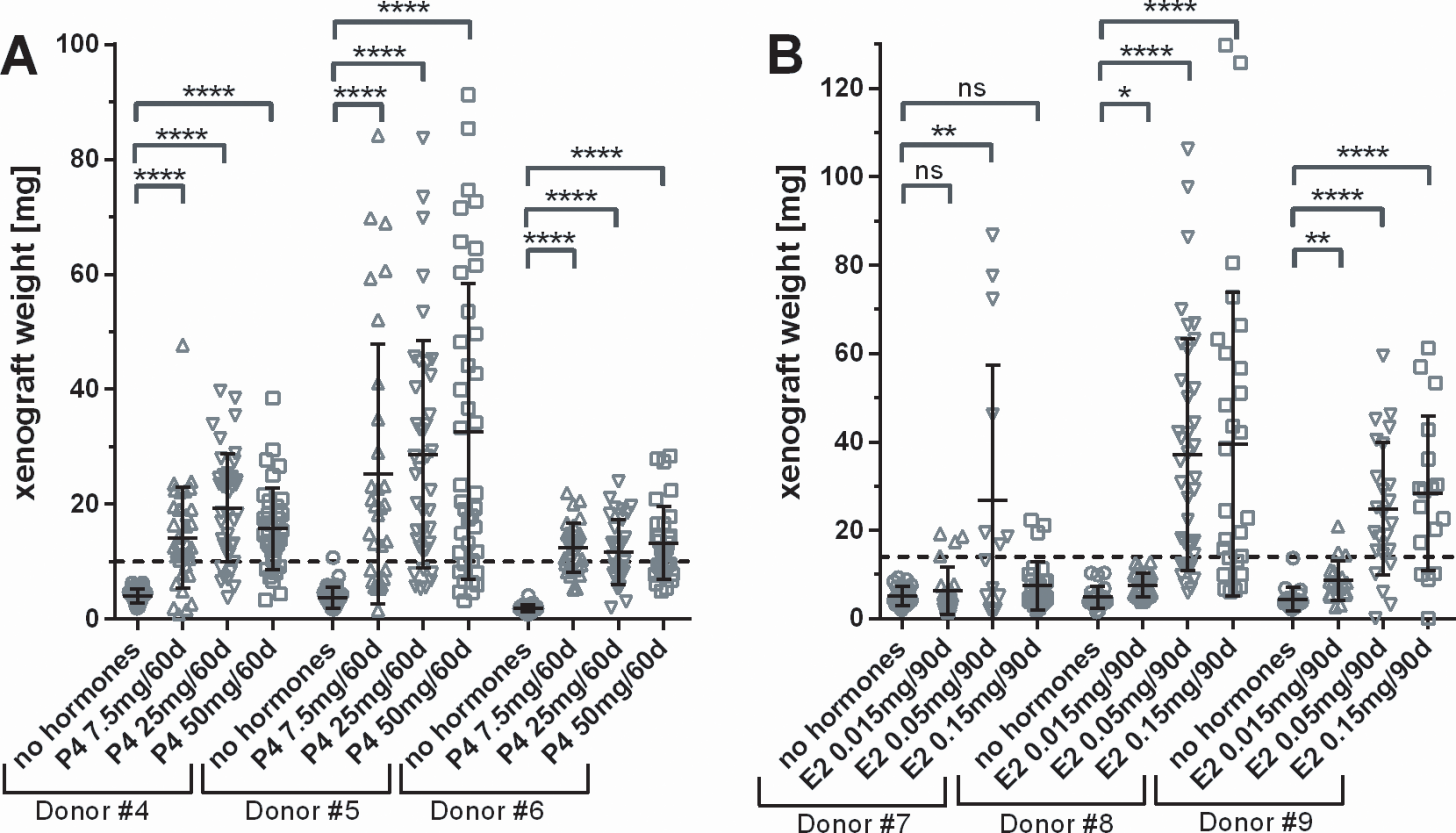
Optimization of 17β Estradiol and Progesterone dose supplement for uterine fibroid xenograft growth. Ovariectomized SCID outbred mice were either not treated or supplemented with Progesterone pellets as indicated, and standard 17β Estradiol pellets (0.05mg/90d release, panel A); or 17β Estradiol pellets as indicated, and standard Progesterone pellets (25mg/60d release, panel B). Small pieces of fibroid tissue where grafted at an average weight indicated with the dotted line. For this experiment, 4 to 5 mice per donor with eight grafts each where used per treatment group. Mice were sacrificed after 60d, and grafts were weighted. Symbols indicate individual graft weights, together with group means ± s.d. Significance of difference between treatment and control groups was evaluated a two-sided t-test with Dunnett´s correction for multiple testing.

Although UF xenografts in mice showed clear growth under E2 and P4 supplementation, high doses of E2 can have untoward effects on the female mouse reproductive tract with severe keratinization of the cervix and vagina and secondary obstruction/infection of the urinary tract. Therefore, we conducted optimization experiments to determine the lowest effective dose of hormones needed to stimulate graft growth. While P4 is essential for graft growth, we found no clear dependency on P4 in the tested dose range; as can be seen in Fig 3A for grafts from donor #4 and donor #5, while tissue from donor #6 did not grow at all. With E2, the fibroid xenograft growth rate shows an optimum with 0.05mg/90d release pellets (Fig 3B). In mice with lower Estradiol doses (0.015 mg/90d release), neither of the tissues from donors #7, #8 or #9 showed any weight gain. With the higher E2 dose (0.15mg/90d release) tissue from donor #8 and #9 showed graft growth similar to the middle Estradiol dose, while tissue from donor #7 remained small. Taken together, the dose of progesterone seems not critical; while optimal graft growth is supported at 0.05mg/90 d release Estradiol doses. Higher doses might again impair graft growth (e.g. donor #7) and lead to unwanted side effects on the female reproductive system. In an attempt to further maximize the growth of fibroid grafts in SCID mice, we assessed graft growth in SCID mouse strains showing different severity of impairment of the immune system. We transplanted tissue from one donor to 4–5 mice per SCID mouse strain and analyzed graft growth after 50 days. Grafts in NOG SCID and Rag2γC mice showed the very same weight gain with material from one donor as ICR SCID mice, while graft weights were lower in NOD SCID mice. In SCID beige, graft growth was up to 50% higher than in the other strains (data not shown).

To compare optimal hormone doses for UF growth found in our experiment with hormone levels in women at different stages of the female cycle, we measured the concentration of E2 and P4 in plasma of the hormone-supplemented mice at the end of the experiment. P4 plasma concentration after 60 d with 7.5, 25, and 50mg/60 release pellets was 3.7±1.9, 9±4, and 21.3±7.4 ng/ml, respectively, while P4 in intact mice is approx. 5ng/ml in proestrous trough, and approx. 35ng/ml in proestrous peak [28], and between 0.3ng/ml and 12ng/ml in the proliferative and secretory cycle of women, respectively [29]. Therefore, supplementation of the mice with 25mg/60d release pellets delivered a P4 plasma level to our mice in the range of the secretory phase of the human female cycle, where strongest fibroid growth can be seen in situ. E2 in mouse plasma at d60 with 0.05mg/90d release pellets was 117±20 pg/ml for CB17-SCID (inbred), 138±48 pg/ml for SCID-beige and 263±78 pg/ml for ICR-SCID (outbred) mice. The E2 concentration in plasma of intact swiss mice was determined to 20 to 60 pg/ml in CD-1, which are most closely related to the SCID mice strains used in our experiments [30], and between 10 to 30 pg/ml in “white swiss strain” intact mice in Metestrous and Proestrous, respectively [28,31]. In women, the range for E2 has been determined to 20–55 pg/ml in proliferative and approximately 80–190 pg/ml in secretory phase [29]. Growth of our fibroid grafts is best with an E2 plasma level in CB17-SCID and SCID beige mice well within the range of women in the secretory phase of the menstrual cycle, where fibroid growth is most prominent.

### Fibroid xenografts are preserving the histology of human uterine fibroids

**Fig 4:**
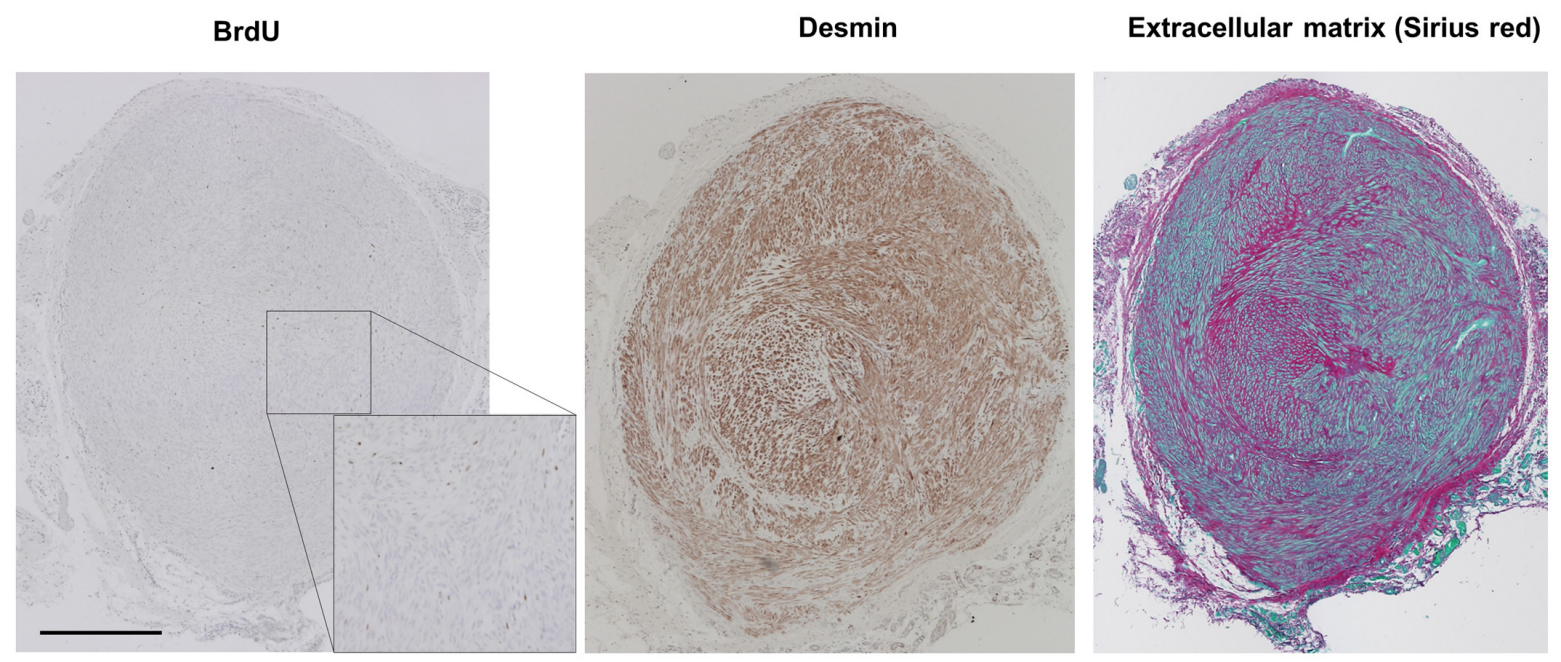
Fibroid grafts retain characteristics of human uterine fibroids. Fibroid xenografts transplanted with approx. 10mg and grown for 60d in SCID mice supplemented with 17β Estradiol and Progesterone to 31mg were stained for BrdU incorporation (left, see S1 Fig for higher resolution), Desmin (middle) and collagen (extracellular matrix) (right). Scale bar = 500μm.

Histologically the grafts were composed of interwoven bundles of smooth muscle cells, and abundant deposition of collagenous material. They showed homogenous morphology between individual grafts in the same as well as in different hosts. These histological characteristics of uterine leiomyoma were preserved for tissues for all donors analyzed. To further characterize whether xenografts also preserve further properties of human uterine fibroid tissue, we stained grafts after passage in mice (Fig 4). Cell nuclei showing BrdU incorporation reflecting the cell proliferation over a 7 day time period were scattered over the graft indicating a homogeneous slow growth. Desmin stain confirmed that the graft thereby preserved the intermediate filament characteristics of smooth muscle cells. Sirius red/fast green stain allows differentiation of cell cytoplasm (green) versus areas of collageneous ECM (red) of the xenografts. Abundant ECM can be seen by Sirius red stain of collagen fibers. By comparison of the Desmin and collagen (ECM) stain, Desmin stain is only seen in regions with cytosol of smooth muscle cells (green in the Sirius red stain).

### Dynymics of Fibroid xenograft growth vary by donor

**Fig 5:**
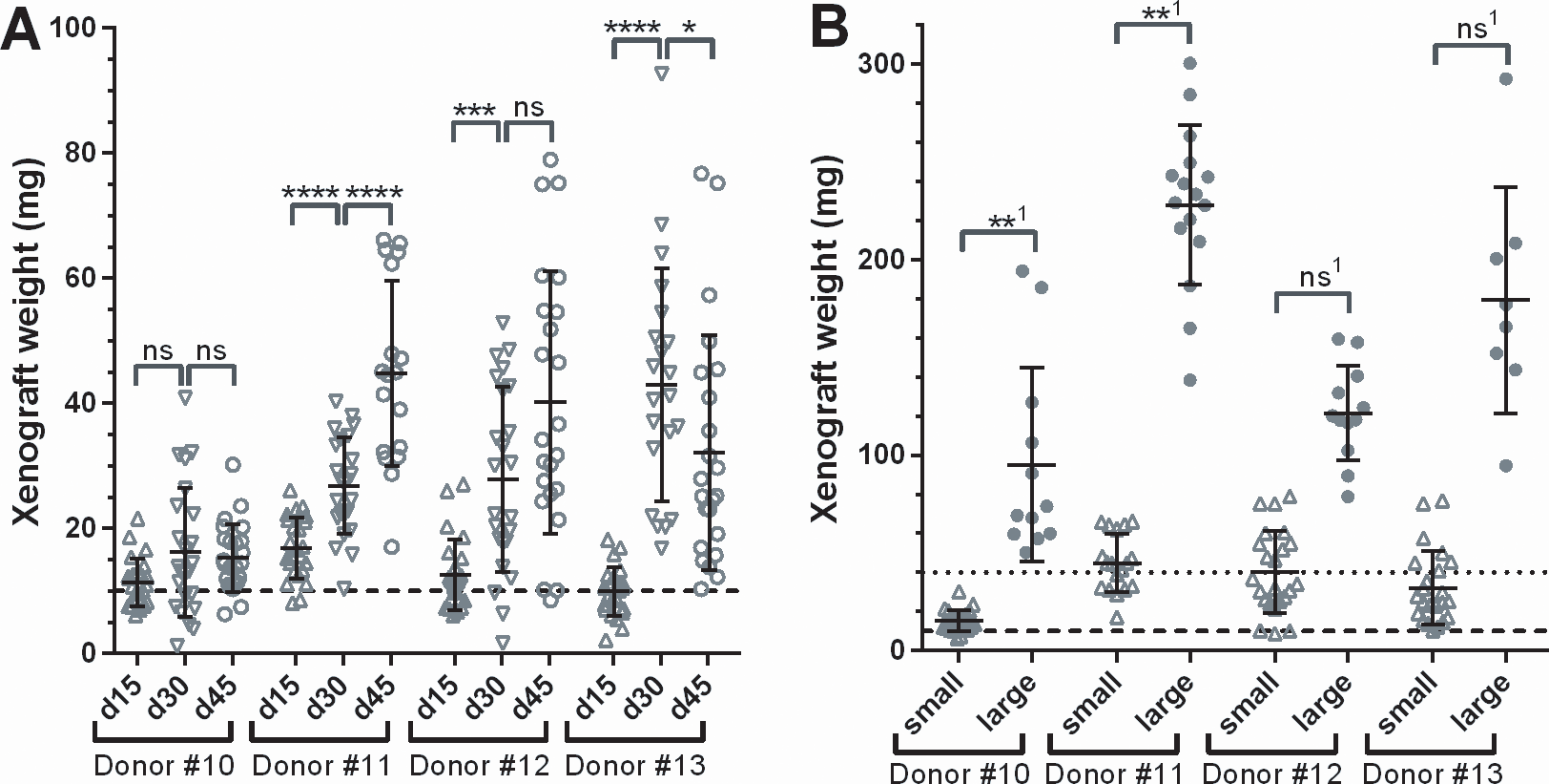
Growth kinetics of human fibroid xenografts in CB17 SCID mice. 17β Estradiol and Progesterone supplemented SCID mice were grafted with small (A) or large (B) grafts with fibroid tissue from four different patients. The dashed and dotted lines indicate graft weights of small and large grafts at transplantation on day 0, respectively. Mice were killed after 15, 30 and 45 days (A) or 45 days (B) and grafts were removed and weighted. Each individual graft weight is shown, together with group means ± s.d. Tissue from donors with the same numbers in panel A and B are identical. For statistical analysis, in (A) log transformed group means d15 to d30, and d30 to d45 were compared. In (B), ^1^graft weights were normalized to the respective xenograft transplantation weight of 10mg for small and 40mg for large grafts, and log transformed normalized data were compared to each other. Significance of difference between treatment and control groups was evaluated using a two-sided t-test.

Clinically, human fibroids show heterogeneous weight gain over time, and may even shrink [32,33]. Therefore, we analyzed the growth kinetic of fibroid xenografts in our SCID mouse model, and also asked whether transplantation of large grafts results in any advantage concerning graft growth dynamic or weight gain (Fig 5A and 5B) compared to smaller grafts. Graft growth showed a lag phase between d0 and d15 during graft establishment. Fibroid tissue from donor#11 and donor#12 showed rather linear growth over time with similar relative weight gain between d15 to d30, and d30 to d45. Grafts from Donor#10 showed only little growth and stalled after d30, while grafts from donor #13 even shrunk from d30 to d45 (panel A). In the same experiment, we also asked whether graft weight gain is dependent on its size at transplantation. Percent graft growth at d45 relative to the graft weight at d0 was very similar for either small (2x2x2mm, ~10mg) or large (2x4x4mm,~40mg) graft pieces of the same donor (Fig 5B). However, the coefficient of variation for the small grafts (35.5%, 33.0%, 52.3% and 58.4% for donors#10 to #13) tended to be higher than those for the large grafts (52.11%, 17.90%, 20.01% and 32.32% for donors#10 to #13 respectively).

### Rapamycin and Mifepristone inhibit xenograft growth

**Fig 6:**
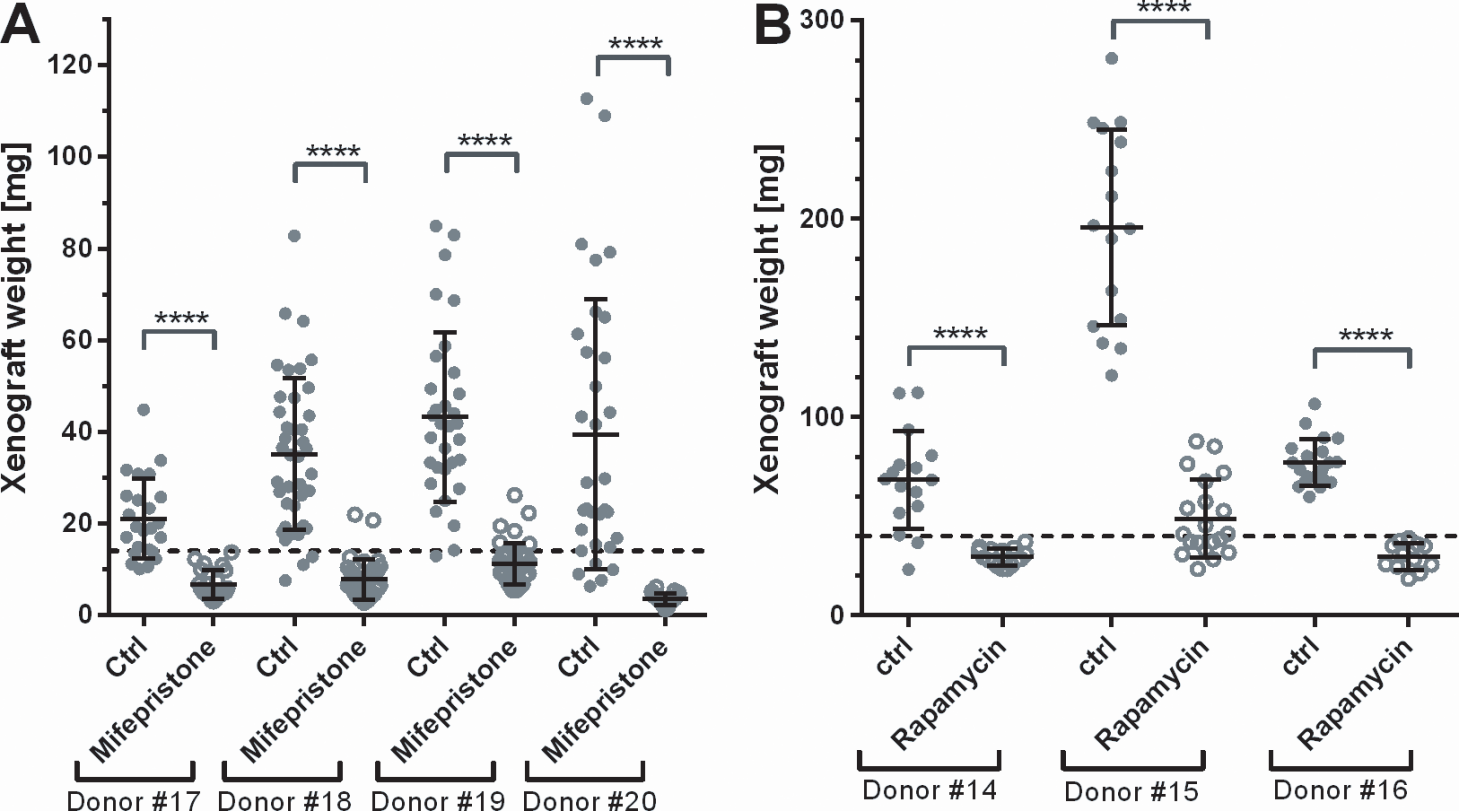
Rapamycin and Mifepristone inhibit growth of fibroid xenografts in SCID mice. E2 and P supplemented SCID-beige mice were grafted with large grafts (for Rapamycin) and small grafts (for Mifepristone, SCID-beige) from fibroid tissue from three and four different patients, respectively. The dashed line indicates the average weight of the grafts at day 0. (A) Mice were treated with Mifepristone from day 0 using 10mg/60d release pellets; this is approx. 6.5mg/kg/d. (B) Mice were treated p.o. with either 15mg/kg/d Rapamycin in 2.5% PEG400 in H_2_O or vehicle alone from d4 of the experiment. Mice were killed after 42–45 days and grafts were removed and weighted. Each symbol indicates the weight of an individual graft, together with group means ± s.d. Significance of difference between treatment and control groups was evaluated using a two-sided t-test.

Because antiprogestins have been shown clinically to reduce the size of uterine fibroids [34], we tested the response of our model to a well characterized antiprogestin, mifepristone. Fig 6A shows that mifepristone treatment completely blocks the growth of the human fibroid grafts and even more led to their shrinkage below their weight at transplantation. We also evaluated the effect of rapamycin, a mTOR pathway inhibitor, which was effective in the EKER rat fibroid model [19]. Results show that human fibroid xenografts in SCID mice from all three donors did not grow under treatment with rapamycin, demonstrating for the first time that rapamycin effectively can inhibit human fibroid growth in an in vivo model (Fig 6B).

## Discussion

In this study we demonstrate for the first time a careful analysis of a successful in vivo xenograft model where human fibroids are grown subcutaneously in immunodeficient SCID mice. Moreover, we show that there is an optimum dose of E2 and P4, respectively, that supports growth of the subcutaneous grafts. Upon completion of this study our take rates approached 100% with material from more than 100 fibroids grafted. Careful tissue selection is the key to successful grafting, e.g. with focus on fibroids being homogeneous, whitish and firm in their structure without signs of necrosis or other degenerations. The grafted tissues maintain the typical characteristics of uterine leiomyomas. Both, ERα and PR remain expressed in the xenografts after 60 days in mice. Expression of both receptors differentiates benign uterine leiomyomas from the rarely occuring malignant leiomyosarcomas [35]. Hormone dependent growth is a hallmark of UFs in women. UFs shrink after menopause due to lack of P4 and reduced E2 levels. Also, antiprogestins have been shown to be efficacious in reducing fibroids in humans. Our model recapitulates that both; E2 and P4 are essential for fibroid xenograft growth, and antiprogestins like mifepristone (this study) and BAY 1002670 [36] completely abolish growth. Here, our in vivo model shows clear advantages over in vitro approaches for studying UFs. Both, in culture of isolated human leiomyoma cells, and in culture of whole tissue pieces preserving the characteristic extracellular matrix, *ESR1* and *PGR* mRNA are strongly downregulated within hours [37,38]. We also demonstrated that, under optimal supplementation with E2 and P4, uterine leiomyoma grafts show reliable proliferation, while myometrial grafts did not grow. This is in accordance to ex vivo studies on proliferation markers in uterine fibroid versus myometrial tissue [25], and in contrast to the slower proliferation of fibroid cells in vitro [39]. Also, uterine leiomyoma in the EKER rat showed much higher proliferation indices in comparison to human UFs, as determined by PCNA stain [26,40]. In conclusion, also with respect to cell proliferation, our in vivo xenograft model much more reflects the in situ situation in comparison to other described models.

As outlined above, the etiology of UFs is essentially not understood. So far, the disease was rarely linked to mutations in a single gene in humans; an example being fumarate hydratase [41]. Mutations in the Tsc gene(s), being drivers of uterine leiomyoma in the EKER rat, have not been linked to the disease in humans. More recent research rather suggests that development of UFs might be mainly driven by either mutations in the *MED12* gene, or by overexpression of the *HMGA2* gene by chromosomal rearrangements [20,42]. The use of patient derived material in our xenograft model is a clear advantage in this respect, since their heterogeneous and unknown etiology is represented in our model, although the grafts present histologically with all criteria for typical uterine leiomyoma. It will be interesting to test whether the different growth capability of the grafts in our hands is related to either *MED12* mutations or *HMGA2* mRNA overexpression.

The histological appearance of the xenografts after 60 days preserves the described histological characteristics of fibroids. The smooth muscle cells of the leiomyoma xenografts do express the smooth muscle marker Desmin. This marker differentiates tumors of smooth muscle cell origin, like leiomyoma or leiomyosarcoma, from those of endometrial origin in the uterus [43]. Sirius red/fast green stain allows differentiation of cell cytoplasm (green) versus areas of collageneous ECM (red) of the xenografts. Excessive deposition collagenous ECM is one hallmark of UFs. Moreover, it is even unclear whether weight gain of UFs is mainly caused by cell proliferation, by cell hypertrophy, or by matrix accumulation. Therefore, treatment of uterine leiomyoma with antifibrotic compounds like pirfenidone might be a successful approach [44]. The described in vivo model allows the evaluation of antifibrotic effects by quantifying matrix in relation to total area in Sirius red stained xenografts. Whole graft histological pictures look quite homogeneous, e.g. one could not distinguish areas of the originally grafted tissue pieces versus newly grown tissue in mice. Growth is not restricted to certain areas of the grafts, but scattered all over the tissue, as can be seen in the histology and by the homogeneous distribution of the BrdU and Ki67-stain. Relative graft growth in the experiments was very much dependent on the individual fibroid tissue employed. Furthermore, statistical analyses showed that tissue source is the main factor for growth variability. Although final graft weights showed large variations even within one treatment group with material from one donor; the individual mouse statistically was not found to have an impact if being from the same strain. Also, the grafts showed rather constant weight gain over time, or even stalled after a period of growth. Again, this recapitulates the described behavior of human fibroids in situ [32,33,45].

It is important to point out that UFs engrafted into these mice do not show exponential growth as can be seen in most malignant tumor xenograft models. Not only does this recapitulate the nature of the fibroids in women, but it also allows for grafting up to eight fibroid tissue pieces per mouse for improved statistics without reaching a total tumor burden critical for animal welfare. SCID beige mice did support graft growth best in our hands, but were more susceptible for side effects known to be induced by high-dose E2 supplement in murines than the (ICR) SCID strain, e.g. urolith formation and subsequent difficulties to control urination [46,47]. NOG SCID and Rag2γC SCID mice strain having an even more compromised immune reaction were also tested for their support of uterine leiomyoma xenograft growth, but did not show a clear advantage compared to the other SCID mice strains. During the development of this model, our tumor take rates constantly improved and approached 100% with material from more than 100 fibroids grafted.

In summary, the established in vivo uterine leiomyoma xenograft model in SCID mice exhibits the hallmarks of UFs growth in situ. Expression of ERα and PR is preserved as well as hormone dependent growth of the tumors. Characteristics of UFs like excessive deposition of extracellular matrix and the whirl-like interwoven organization of the smooth muscle cells are also maintained, even in newly grown areas. Fibroid xenografts are responsive to medical treatment modalities like rapamycin and mifepristone. The xenograft model presented here allows testing and validation of new approaches and drugs to treat human uterine fibroids. It is suited to test hypotheses on the development of the disease by interfering with specific pathways to see whether they have an impact on graft growth or extracellular matrix deposition. Furthermore, it allows routine testing of drug candidates in preclinical drug discovery. As a major strength the growth of the patient-derived xenografts is not an artificial model driven by single mutations suspected to be a cause for the disease, but reflect the heterogeneity of the human fibroid tumor etiology. It therefore represents an unbiased approach towards the identification of new treatment options. However, due to the subcutaneous location of the grafts we cannot exclude that the lack of interaction of the fibroid tissue with its normal surrounding tissue, e.g. the endometrium, does restrict its full physiological function and response. Orthotopic transplantation of the human fibroid tissue pieces into the uterus of SCID mice might therefore further improve this model.

## Supporting Information

S1 Table Detailed information on tissue donor characteristics and mice strain and number used for each experiment. (DOCX)

S1 Fig High resolution figure of BrdU stain in leiomyoma xenograft in Fig 4. (TIF)
