# Supplementary material for: Application of a Patient Derived Xenograft Model for Predicative Study of Uterine Fibroid Disease
Source: PLoS One. 2015 Nov 20;10(11):e0142429. doi: 10.1371/journal.pone.0142429 (PMC4654507; doi:10.1371/journal.pone.0142429)
Supplement: S1 Table — (DOCX) [file pone.0142429.s001.docx]

**S1 Table. Tissue donor and mouse xenograft experiment detailed data**

|  |  |  | Donor data | | | |  | Mouse xenograft experiment data | | | |
| --- | --- | --- | --- | --- | --- | --- | --- | --- | --- | --- | --- |
| Tissue Donor | Fig |  | Donor Age | days since last menses | Diagnosis | Hormone treatment |  | Mouse strain | Graft size | Grafts/mice transplanted per group | Grafts/mice recovered  per group |
| 1 | 1A/B |  | n.a. | 23 | Uterus myomatosus | no |  | CB17-SCID | small | 40/5, 32/4, 32/4 ^1^ | 30/5, 29/4, 30/4 |
| 2 | 1A/B |  | n.a. | 26 | Uterus myomatosus | no |  | CB17-SCID | small | 24/3, 24/3, 24/3 ^1^ | 20/3, 18/3, 20/3 |
| 3 | 1A/B |  | n.a. | 11 | Uterus myomatosus | no |  | CB17-SCID | small | 24/3, 40,5, 48/6 ^1^ | 23/4, 24/5, 25/6 |
| 4 | 3A |  | 50 | 30 | Uterus myomatosus | no |  | ICR-SCID | small | 32/4, 32/4, 32/4, 32/4 | 24/4, 24/4, 14/3, 27/4 |
| 5 | 3A |  | 42 | n.a. | Uterus myomatosus | n.a. |  | ICR-SCID | small | 32/4, 32/4, 40/5, 32/4 | 22/3, 28/4, 39/5, 27/4 |
| 6 | 3A |  | 39 | 21 | Uterus myomatosus | no |  | ICR-SCID | small | 24/3, 24/3, 32/4, 32/4 | 19/3, 18/3, 24/4, 17/3 |
| 7 | 3B |  | 41 | 17 | Uterus myomatosus | no |  | ICR-SCID | small | 40/5, 40/5, 40/5, 40/5 | 36/5, 35/5, 40/5, 39/5 |
| 8 | 3B |  | 48 | 21 | Uterus myomatosus | n.a. |  | ICR-SCID | small | 40/5, 40/5, 40/5, 40/5 | 37/5, 31/4, 40/5, 40/5 |
| 9 | 3B |  | 31 | 55 | Uterus myomatosus | no |  | ICR-SCID | small | 32/4, 32/4, 32/4, 32/4 | 31/4, 32/4, 32/4, 32/4 |
| 10 | 5A |  | 44 | 30 | Uterus myomatosus | no |  | CB17-SCID | small | 24/4, 24/4, 24/4 | 24/4, 24/4, 24/4 |
|  | 5B |  |  |  |  |  |  | CB17-SCID | small/large | 24/4, 16/4 | 24/4, 12/3 |
| 11 | 5A |  | 44 | 10 | Uterus myomatosus | - |  | CB17-SCID | small | 24/4, 24/4, 24/4 | 24/4, 24/4, 18/3 |
|  | 5B |  |  |  |  |  |  | CB17-SCID | small/large | 24/4, 16/4 | 24/4, 16/4 |
| 12 | 5A |  | 48 | 7 | Uterus myomatosus | no |  | CB17-SCID | small | 24/4, 24/4, 24/4 | 24/4, 24/4, 23/4 |
|  | 5B |  |  |  |  |  |  | CB17-SCID | small/large | 24/4, 16/4 | 24/4, 12/3 |
| 13 | 5A |  | 46 | 9 | Uterus myomatosus | EE/LNG |  | CB17-SCID | small | 24/4, 24/4, 24/4 | 24/4, 24/4, 24/4 |
|  | 5B |  |  |  |  |  |  | CB17-SCID | small/large | 24/4, 16/4 | 24/4, 8/2 |
| 14 | 6B |  | 48 | 8 | Uterus myomatosus | No |  | SCID-beige | large | 20/5, 20/5 | 16/4, 16/4 |
| 15 | 6B |  | 44 | 14 | Uterus myomatosus | no |  | SCID-beige | large | 20/5, 20/5 | 16/4, 20/5 |
| 16 | 6B |  | 42 | continuous bleeding | Uterus myomatosus | LNG IUD |  | SCID-beige | large | 20/5, 20/5 | 20/5, 12/3 |
| 17 | 6A |  | 40 | 16 | Uterus myomatosus | no |  | SCID-beige | small | 24/3, 24/3 | 24/3, 23/3 |
| 18 | 6A |  | 49 | 22 | Uterus myomatosus | no |  | SCID-beige | small | 40/5, 40/5 | 40/5, 36/5 |
| 19 | 6A |  | 31 | 22 | Uterus myomatosus | no |  | SCID-beige | small | 40/5, 40/5 | 32/4, 40/5 |
| 20 | 6A |  | 48 | 12 | Uterus myomatosus | no |  | SCID-beige | small | 32/4, 32/4 | 32/4, 24/3 |

^1^ In this experiment four fibroid and four myometrial grafts were transplanted per each mouse
