# Supplementary figures and images for: Application of a Patient Derived Xenograft Model for Predicative Study of Uterine Fibroid Disease
Source: PLoS One. 2015 Nov 20;10(11):e0142429. doi: 10.1371/journal.pone.0142429 (PMC4654507; doi:10.1371/journal.pone.0142429)

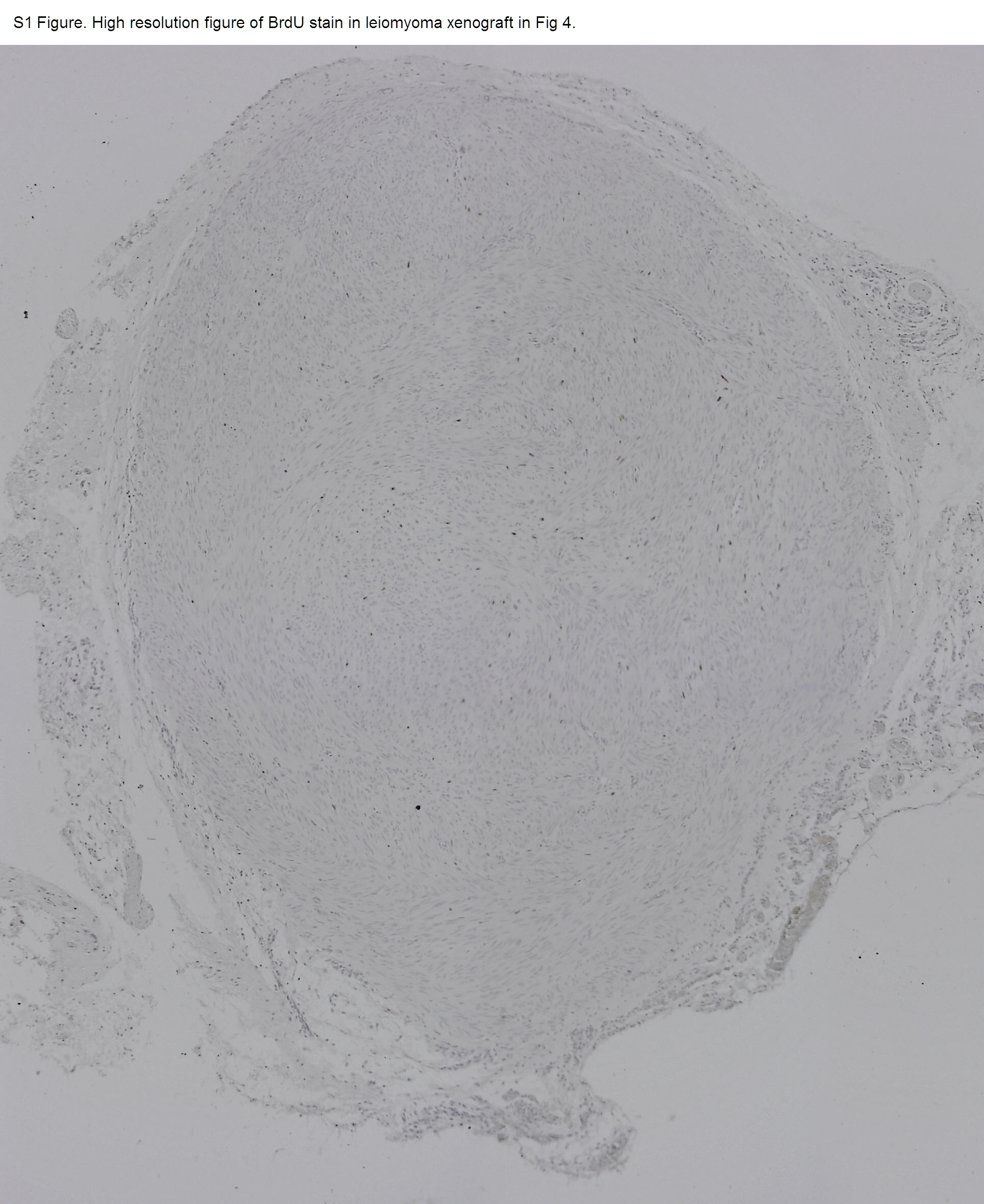

Supplement: S1 Fig — (TIF) [file pone.0142429.s002.tif]
